# Knowledge of Obstetric Fistula Prevention amongst Young Women in Urban and Rural Burkina Faso: A Cross-Sectional Study

**DOI:** 10.1371/journal.pone.0085921

**Published:** 2013-12-31

**Authors:** Aduragbemi O. Banke-Thomas, Salam F. Kouraogo, Aboubacar Siribie, Henock B. Taddese, Judith E. Mueller

**Affiliations:** 1 Centre for Maternal and Newborn Health, Liverpool School of Tropical Medicine, Liverpool, Merseyside, United Kingdom; 2 Epidemiology and Bio-statistics Department, École des Hautes Études en Santé Publique, Rennes, Bretagne, France; 3 Surgery department, District Hospital (Centre Médical avec Antenne chirurgicale), Boromo, Boucle du Mouhoun, Burkina Faso; 4 School of Health and Related Research, the University of Sheffield, Sheffield, South Yorkshire, United Kingdom; International AIDS Vaccine Initiative, United States of America

## Abstract

Obstetric fistula is a sequela of complicated labour, which, if untreated, leaves women handicapped and socially excluded. In Burkina Faso, incidence of obstetric fistula is 6/10,000 cases amongst gynaecological patients, with more patients affected in rural areas. This study aims to evaluate knowledge on obstetric fistula among young women in a health district of Burkina Faso, comparing rural and urban communities. This cross-sectional study employed multi-stage sampling to include 121 women aged 18-20 years residing in urban and rural communities of Boromo health district. Descriptive statistics and multiple logistic regression analysis were used to compare differences between the groups and to identify predictors of observed knowledge levels. Rural women were more likely to be married (*p*<0.000) and had higher propensity to teenage pregnancy (*p*=0.006). The survey showed overall poor obstetric fistula awareness (36%). Rural residents were less likely to have adequate preventive knowledge than urban residents [OR=0.35 (95%-CI, 0.16–0.79)]. This effect was only slightly explained by lack of education [OR=0.41 (95%-CI, 0.18–0.93)] and only slightly underestimated due to previous pregnancy [OR=0.27 (95%-CI, 0.09–0.79)]. Media were the most popular source of awareness amongst urban young women in contrast to their rural counterparts (68% vs. 23%). Most rural young women became ‘aware’ through word-of-mouth (68% vs. 14%). All participants agreed that the hospital was safer for emergency obstetric care, but only 11.0% believed they could face pregnancy complications that would require emergency treatment. There is urgent need to increase emphasis on neglected health messages such as the risks of obstetric fistula. In this respect, obstetric fistula prevention programs need to be adapted to local contexts, whether urban or rural, and multi-sectoral efforts need to be exerted to maximise use of other sectoral resources and platforms, including existing routine health services and schools, to ensure sustainability of health literacy efforts.

## Introduction

Obstetric Fistula is defined as a direct communication between the vagina and the bladder (vesico-vaginal fistula) and/or between the vagina and the rectum (recto-vaginal fistula) [1]. It usually occurs after prolonged or obstructed labour, spontaneous abortion or female genital mutilation and leads to physiological anomalies, including continuous loss of urine or faeces through the vagina, urogenital infections, inflammation of the skin (dermatitis ammonia), kidney infections and is usually accompanied by other manifestations from the “obstructed labour injury complex” including damage to the cervix or pelvic bones, foot drop, genital lacerations, and amenorrhea (absence of menstruation). In addition, fistula patients face high levels of stigma from their families and communities resulting in social and economic exclusions, which in turn lead to high risks of depression and suicide [2–4]. 

In sub-Saharan Africa and South Asia, obstetric fistula is very common, as access to and use of emergency obstetric care is limited [5]. While it is widely accepted that it is difficult to estimate the incidence and prevalence rates of obstetric fistula in developing countries, it is estimated that more than 2 million young women live with untreated obstetric fistula in Asia and sub-Saharan Africa [6–8]. Some studies have suggested incidence rates to be between 1 and 3 per 1000 births in West Africa [8], with higher rates of 5 to 10 cases in 1000 births reported in rural areas of sub-Saharan Africa [9]. Several factors have been linked to the high occurrence of obstetric fistula in sub-Saharan Africa, including the preponderance of early marriage and teenage pregnancy, which in turn mean that the girls do not have pelvises which have sufficiently developed to allow reproduction [10]. This is further compounded by the poor nutritional status of most of the girls who live in these highly deprived settings [11,12]. 

In Burkina Faso, obstetric fistula remains a huge problem [5]. According to the statistical yearbook of the Department of Studies and Planning, the incidence of obstetric fistula in Burkina Faso was estimated at 6/10,000 cases at gynaecological outpatient clinics for the year 2000 [13]. In addition, several more fistula patients do not present at the facilities, because the condition usually affects the most marginalized group, that is, poor, young women who are often illiterate and who live in rural areas [6]. The median age of women with fistula is 25 years and 96.3% of the women are housewives without their own income [14]. Regarding the cost of treatment, the budget presented by Dédougou Regional Hospital to UNFPA (United Nations Population Fund) for the last obstetric fistula surgical mission in November 2011 reported a total cost of 238,658 FCFA (equivalent to US$  480) for the surgical treatment of each obstetric fistula patient [15].

Burkina Faso is a francophone West Africa country with an economy that is mainly based on agriculture and livestock [16]. It has an estimated population of 13,730,258 inhabitants and an annual population growth rate of 3.1%, whereby women represent approximately 51.8% of a largely young population; 47.9% of the total population are within the 0 - 14 years of age bracket [16]. About 78% of the population lives in rural areas and women of childbearing age (15 - 49 years) constitute 24% of the total population [16,17]. There is also a relatively high early fertility (130 ‰ at 15 - 19), which increases rapidly to a peak at 25 - 29 years (269 ‰) [17]. Burkina Faso is a low-income country, whereby about 45.3% of the population live below the poverty line and the income per capita is estimated at just 72,690 FCFA (approximately US$  146) per person per year. Women bear the burden of the poverty [16].

Currently, there is an on-going 4-year program, covering the period, 2011 - 2015, to eradicate obstetric fistula from Burkina Faso [18]. However, the task of eradicating this condition remains daunting [19]. This research was conducted in Boromo health district, which is in the Boucle du Mouhoun region of Burkina Faso. With support from international non-governmental organizations, 76 cases of obstetric fistula were treated ​​within the district between October 2009 and December 2011 [20]. The region has one of the highest fertility rates in Burkina Faso and harmful practices such as female genital mutilation, early marriage and wife inheritance persist throughout the region despite repeated campaigns [21]. This study aims to assess the prevalence of knowledge on obstetric fistula amongst the population of young women in both rural and urban communities of the Boucle du Mouhoun region.

## Materials and Methods

### Ethics Statement

Ethics approval for this study was obtained from the ethics committee of the School of Health and Related Research (ScHARR), University of Sheffield, in the UK, as well as Ecole des Hautes Études en Santé Publique (EHESP), in France, and the National Centre for Scientific and Technological Research (CNRST), and the National Health Research Ethics Committee (CERS), in Burkina Faso. Participants’ written informed consent was obtained using an informed consent form, which had been reviewed and approved by all four ethics committees. For those participants who could not read and write, the enumerators read out the research information sheet and the informed consent form, and their thumbprint was taken as proof of consent.

### Methods

This is a descriptive cross-sectional study, which assesses and compares knowledge of young women (aged 18-20 years) on obstetric fistula in urban and rural areas of Boromo health district. The survey was conducted between May 15, 2013 and May 19, 2013. We employed a cluster sampling method, which included two groups: the only urban area and 3 out of the 9 rural areas in Boromo. The sample size was estimated using STATA SE 12.1, targeting a power of 80% and an α error of 0.05 for the comparison of population level knowledge in both groups (rural Boromo and urban Boromo). Ideally, the knowledge should be 100% widespread, but in this study, we made an assumption that 80% knowledge prevalence was "sufficient" to effectively propagate any piece of information that needs to be disseminated. The sample size was calculated with the intention of being able to describe a 20% significant difference in knowledge, that is, an observed prevalence of 60% or lower. These two proportions were used to generate the sample size, with a design effect (D_eff_) of 2. The assumption made to estimate "ρ" clusters was similar within each sample, to bring the value to "1". The computation under these assumptions prescribed a sample size of 24 for each group (urban and rural), which adds to 48 for the whole study. Subsequently, factoring in the design effect led to an estimation of a sample size of 96. The method used in populating the groups had been utilized previously in studies of prevalence of meningococcal carriage in rural and urban communities of Bobo-Dioulasso, Burkina Faso [22). The multi-stage sampling entailed that streets or departments would be selected purposely while compounds or villages would be identified by systematic random sampling method. This sampling technique was preferred as it was found to be more feasible and as it allowed enumerators to work more efficiently [23]. Details of the sampling procedure are presented below:

For urban Boromo,

First stage: In the urban area, 3 streets were selected randomly beginning with a randomly identified starting point. This was done after reviewing all streets on the cadastral plans. 

Second stage: Identification of compounds to be included in the survey was done by spinning a pen on a notebook and progressing in the direction indicated by the tip and entering every second compound that was encountered during the walk. If no eligible young woman, who met the inclusion criteria, was found within the compound, the investigators continued to the next compound. In each compound, the investigators included only one person, who was in turn identified randomly.

For rural Boromo,

First stage: To start with, 3 of the 9 administrative departments were identified randomly. 

Second stage: From the principal departments, 50 hamlets were identified through systematic random sampling. The villages to be included in the survey were identified by spinning a pen on a notebook and moving in the direction indicated by the tip of the pen, entering every second village encountered. One eligible woman was included per hamlets. A hamlet in this setting typically represents small settlements of around 7-10 households’. 

In each compound (urban Boromo) and hamlet (rural Boromo), a resident young woman of Burkinabe origin, who is between 18 and 20 years, who had not suffered or is currently not suffering from obstetric fistula, was randomly selected and recruited into the study. The eligible age range was set between 18 and 20 years following the rationale that assessing the prevalence of knowledge in a young group would have important ramifications, as they constitute the most affected age group and as they present with opportunities for early intervention with prevention messages. However, we limited the lower age bracket to 18 years as going below that would entail including children in this sensitive subject area, which would in turn lead to quite strenuous and time consuming processes in terms of obtaining ethics approval from the ethics approval boards of the different institutions involved in the research, as well as the host country. 

Female interviewers administered the designed semi-structured questionnaires to assess the knowledge of participants on obstetric fistula. The analysis compared knowledge in rural and urban areas of Boromo. A pretesting of the questionnaire was conducted prior to the main study using 5 volunteers. These volunteers were excluded from the main study. Following the pilot study, the necessary modifications were made to the instrument ​​to simplify the language and facilitate comprehension on the part of participants. The questionnaire was written in French, and the most common local languages Mossi and Dioula. All versions were tested as part of the pre testing. Female research assistants who were trained on the administration of the instrument and who had experience in previous researches were involved in administering the questionnaire.

For the construction of the questionnaire, we consulted previous attempts at assessing knowledge in the area [24], albeit for patients rather than the general population. We also drew upon our understanding of the critical etiological and programmatic considerations in the area. Accordingly, the first section of the questionnaire collected information on the socio-demographic background of the respondents including age, level of education, marital status, age at first marriage, number of previous pregnancies and age of first pregnancy (for those who reported being pregnant previously). The second section of the questionnaire inquired about the awareness of respondents about obstetric fistula. “Awareness” in this study simply referred to whether the respondent had previously heard about obstetric fistula. For those respondents who answered, “Yes”, they were classified as being “aware”, while those who responded “No”, were classified as “unaware”. 

The third section of the questionnaire was used to assess participants' knowledge. "Knowledge" in this study was defined as "the knowledge of risk factors and symptoms of obstetric fistula, normal duration of labour and possible sources of emergency obstetric care” (Table 1). An assumption was made in this study that knowledge of these four critically important aspects would contribute towards the reduction of the incidence and prevalence of obstetric fistula. All aspects were given equal weight. To test for knowledge of risk factors, we put forth a range of inaccurate propositions (evil spirits, bad luck, prohibited act committed by a woman) and a set of accurate ones (female genital mutilation, home delivery, prolonged labour and malnutrition) (Table 1). We applied the same multiple-choice format to inquire about respondents’ knowledge on symptoms of obstetric fistula. Inaccurate propositions that were put forward in this regard included continuous sleeping and stomach ache, while urinary incontinence, faecal incontinence and vulvar irritation constituted the accurate ones (Table 1). On the other hand, we adopted a ‘Yes’ or ‘No’ response format to assess knowledge on duration of labour and possible sources of emergency obstetric care. Participants who scored 50% or more (providing correct answers to two or more of the questions) were classified as "sufficiently informed" while those with a score of less than 50% were classified as "insufficiently informed". The percentage of "informed participants" was the "prevalence of knowledge". Based on their responses, adolescents were classified into one of these two categories.

**Table 1:** Questions asked to test knowledge.

**Aspects**	**Score/question**
**1. Risk factors associated with obstetric fistula**	**1**
- Early pregnancy	0.125
- Home delivery	0.125
- Female Genital Mutilation	0.125
- Evil spirits	0.125
- Breach of a prohibited act	0.125
- Prolonged labour	0.125
- Bad luck	0.125
- Malnutrition of the mother	0.125
**2. Symptoms of obstetric fistula**	**1**
- Urinary incontinence	0.2
- Faecal incontinence	0.2
- Continuous sleeping	0.2
- Stomach ache	0.2
- Vulva irritation	0.2
**3. Duration of normal labour**	**1**
**4. Source of emergency obstetric care**	**1**
**Total**	**4**

We used standard statistical methods for the analyses, including accounting for design effect. Participant characteristics were compared between the rural and urban group using Fisher’s exact test for categorical and Wilcoxon ranksum test for continuous variables. Odds ratios for the association between rural residency and awareness or knowledge about obstetrical fistula were calculated using logistic regression. To evaluate whether characteristics differing between rural and urban participants (educational status, previous pregnancy and marital status) could explain or had masked an association between rural residency and the outcomes, we calculated adjusted odds ratios. To evaluate whether educational status or previous pregnancy influenced the association between rural residency and outcomes, we also calculated stratified odds ratios. All analyses were performed using STATA ™ SE 12.1. 

## Results

In total, 126 contacts were made, whereby 5 declined to participate, making the total number of respondents to be 121. Of the 5 who declined to participate, 3 were from the urban group and 2 from the rural group. The median age of participants was 19 years old. Around half of participants (50.4%) in the total sample had not received school education, 45.5% were married and 45.5% had been pregnant in the past (regardless of outcome) (Table 2). Age at first pregnancy varied between 15 to 20 years. Participants residing in rural areas (62.0% of total) were significantly less educated, had more frequently experienced pregnancy and had more women who are married, than their urban counterparts [Table 2]. There was no divorced or widowed participant. 

**Table 2:** Characteristics of participants included in the survey.

		**Total (N=121)**	**Rural Boromo (N=58)**	**Urban Boromo (N=63)**	*P* for difference between rural and urban *
Age	Mean (SD)	19.0 (0.9)	19.1 (0.9)	18.9 (0.9)	0.352
Level of education	Non-educated	61 (50.4%)	36 (62.0%)	25 (39.7%)	0.004
	Primary	21 (17.4%)	12 (20.7%)	9 (14.3%)	
	Secondary	39 (32.2%)	10 (17.2%)	29 (46.0%)	
Marital status	Married	55 (45.5%)	38 (65.5%)	17 (27.0%)	0.000
	Single	66 (54.5%)	20 (34.5%)	46 (73.0%)	
Previous pregnancy	Yes	55 (45.5%)	34 (58.6%)	21 (33.3%)	0.006
Age at first pregnancy (years)	Median (min, max)	18.0 (15, 20)	17.5 (15, 20)	18.0 (15, 20)	0.894
Number of pregnancies	Median (min, max)	1 (1, 3)	1 (1, 3)	(1, 3)	0.479

Women aged 18 to 20 years in Boromo district, Burkina Faso, 2013.

^*^ Fisher exact or Wilcoxon ranksum test

Only a third of participants were aware of obstetric fistula, with marginal difference between rural (37.9%) and urban residents (34.9%), which was not biased by educational status, experience of pregnancy [Table 3] or marital status (data not shown).

**Table 3:** Awareness and knowledge on obstetric fistula and effect of rural residency to these outcomes.

		**Total (N=121)**	**Rural Boromo (N=58)**	**Urban Boromo (N=63)**	**Crude OR (95%-CI**)	**OR (95%-CI) adjusted for education**	**OR (95%-CI) adjusted for previous pregnancy**
Awareness of obstetric fistula	Yes	44 (36.4%)	22 (37.9%)	22 (34.9%)	1.14 (0.47, 2.76)	1.69 (0.61, 4.70)	1.06 (0.43, 2.62)
Source of awareness	Word of mouth	18 (40.9)	15 (68.2)	3 (13.6)			
	School	6 (13.6)	2 (9.1)	4 (18.2)			
	Media	20 (45.5)	5 (22.7)	15 (68.2)	0.14 (0.04, 0.46) *	0.12 (0.02, 0.81)	0.13 (0.03, 0.61)
Knowledge of obstetric fistula	Informed	44 (36.4%)	14 (24.1%)	30 (47.6%)	0.35 (0.16, 0.79)	0.41 (0.18, 0.93)	0.27 (0.09, 0.79)
Awareness of risk of complications during delivery	Yes	13 (10.7%)	10 (17.2%)	3 (4.8%)	4.17 (1.63, 10.66)	3.95 (1.41, 11.07)	4.11 (1.18, 14.31)
Knowledge of transport means in case of emergency	Ambulance	19 (15.7%)	7 (12.1%)	12 (19.0%)	0.58 (0.24, 1.44) **	0.74 (0.31, 1.72)	0.87 (0.32, 1.41)
	Motorcycle	87 (71.9%)	41 (71.7%)	46 (73.0%)			
	Donkey	1 (0.82%)	1 (1.7%)	0 (0.0%)			
	Foot	14 (11.6%)	9 (15.5%)	5 (7.9%)			

Women aged 18 to 20 years in Boromo district, Burkina Faso, 2013.

Figures present N (%); OR, odds ratio

* OR for having had information on obstetric fistula by media, vs. word of mouth or school

** OR for mentioning ambulance as the means of transport in case of emergency, vs. other means.

Regarding sources of information, most of the women who were aware of obstetric fistula got the information through the media (45.5%) or attributed the information to family and friends (word of mouth, (41.0%), with predominance of word of mouth among rural women (68.2%). Compared to urban women, and irrespectively of education status, previous pregnancy or marital status, rural women were about 8 times less likely to have received information through the media. 

More participants from urban Boromo were classified as ‘sufficiently informed’ on obstetric fistula (47.6% vs. 24.1%)[Table 3]. Women in rural areas were three times less likely (OR 0.35, CI-95%, 0.16, 0.79) to demonstrate sufficient knowledge, and this effect was only slightly explained by lack of education and only slightly underestimated by previous pregnancy (see adjusted odds ratios in Table 3). 

In stratified analyses using the adjusted model (Table 4), the negative association between rural residence and knowledge of obstetric fistula was particularly strong among women without school education (OR 0.17; 95% CI, 0.05, 0.55), but attenuated and no longer significant among women with any schooling (OR 0.45; 95% CI, 0.13, 1.56). Among women with previous experience of pregnancy, rural residency was even more strongly associated with lack of knowledge than among women without. Sufficient knowledge on obstetric fistula tended to be more common among women with previous pregnancy than without: 66.7% vs. 38.1% among urban residents, and 26.5% vs. 20.8% among rural residents (differences not significant). Inclusion of marital status as covariate did not impact any of the effect estimates but weakened the model due to limited sample size (data not shown).

**Table 4:** Association between of rural residency and knowledge on obstetric fistula by group, by level of education.

	**Crude Odds Ratio** (**95%-CI**)	**Odds Ratio (95%-CI) adjusted for education and/or previous pregnancy**
Overall (N=121)	0.35 (0.16, 0.79)	0.31 (0.10, 0.92)
Non educated (N=61)	0.26 (0.11, 0.64)	0.17 (0.05, 0.55)
Primary education (N=21)	0.67 (0.15, 2.89)	0.69 (0.20, 2.43)
Secondary education (N=39)	0.62 (0.08, 4.94)	0.57 (0.05, 5.96)
Previous pregnancy (N=55)	0.18 (0.05, 0.67)	0.20 (0.05, 0.71)
No previous pregnancy (N=66)	0.43 (0.11, 1.68)	0.51 (0.13, 2.06)

Women aged 18 to 20 years in Boromo district, Burkina Faso, 2013.

All 121 participants identified the hospital as a site where they could access emergency obstetric care, but only 11.0% of women thought they could have pregnancy complications that would require emergency treatment (Table 3). Rural residents were four times more likely to exhibit this understanding (OR 4.17, 95% CI, 1.63, 10.66), irrespective of education or previous pregnancy.

Most women (71.7%) identified the motorcycle and only 15.7% the ambulance as the means for transport in case of emergency, with no significant association with rural residency (Table 3). 

## Discussion

The results of this survey among young women in Burkina Faso suggest that the prevalence of awareness of obstetric fistula and knowledge on prevention are low in both urban and rural areas. From the results, women in rural areas were almost three times less likely to have preventive knowledge on obstetric fistula compared to urban women, and these differences could not be explained by differences in education, experience of previous pregnancy or marital status. A similar cross-sectional study on knowledge of women on antenatal care conducted in Alexandria, Egypt, also showed that urban women had a higher mean total score for antenatal care knowledge than their rural counterparts, with a statistically significant difference (11.23 +/- 2.91 and 6.59 +/- 4.14, respectively and Z = 9.73, P < 0.001) [25]. This urban - rural knowledge gap signifies the need for focusing on rural women with targeted messages on maternal health generally, and on the risks and consequences of fistula and ways of preventing it, specifically. 

Higher level of education amongst urban young girls could explain their higher health literacy, as more young urban women speak and understand the French language, even though there are other factors that could contribute to this rural-urban literacy gap [26]. This could have a massive impact on the amount of information that rural young women can access, as French is the lingua franca and the most spoken language for health media programs, in contrast to the local dialects. Our results from stratifying for education suggest that schooling, even basic primary education, can reduce the rural-urban gap in knowledge related to obstetric fistula, and ultimately contribute to prevention of its occurrence. The education gradient has long been known to be associated with better health behaviours and improved health status [27] and our analysis highlighted this with respect to knowledge of obstetric fistula. In another study that was conducted in Shanghai, education was found to be the most important determinant of maternal health knowledge amongst rural-to-urban migrant women [28]. Furthermore, it has been shown that schools provide an important medium for propagating health education, especially sexual health education. A program implemented in 62 primary schools in rural Mwanza, Tanzania, based on a curriculum which was teacher-led and peer-assisted, showed improved knowledge of risks and benefits of behaviours [29]. 

It is now widely accepted that keeping girls in schools, especially, ensuring that they complete at least primary education, contributes to women empowerment, curtails harmful traditional practices such as child marriage, promotes gender equality and reduces incidences of maternal morbidity and mortality, including obstetric fistula [30,31]. It follows this understanding that there are strong calls for strengthening inter-sectoral collaboration between the ministries of health and education. A working WHO document emphasizes this need to promote multi-sectoral linkages between ministries of health, education and social protection, if improvements are to be made on the state of maternal health, globally [32]. Specifically, there is need to revamp sex education as a whole in the curriculum. A qualitative study conducted in Burkina Faso in 1993 recommended that sex education should be introduced earlier than the beginning of high school and that the program should include discussions on social norms and beliefs about contraception and their modes of action, their advantages and disadvantages, fear of infertility, and the choice of partner [33]. In addition to these suggestions, based on findings from our research, there is need to include discussions on the myriad of complications during early marriage and early pregnancy, including obstetric fistula. This would ensure that young women are already empowered with critical information to aid their decision-making when they get pregnant.

Those young women who were ‘aware’ of obstetric fistula in rural areas mostly attributed their awareness to their family and friends, that is, through word of mouth. In this regard, it is difficult to ascertain the authenticity and quality of the information they receive, in contrast to structured and targeted messages from health professionals, counsellors or through the media. A recent systematic review and meta-analysis demonstrated the positive effects of face-to-face tailored messages from health workers on health behaviours of participants [34]. Similarly to our study, another study carried out in Tanzania showed that young rural women have limited access to mass media when compared to their urban counterparts and that even though word of mouth communication between family and friends plays a large role in the spread of news and information, its role in spreading family planning, and other maternal and child health information is low [35]. Hence, there is need to employ innovative approaches in order to ensure propagation of targeted, structured and accurate information amongst rural young women, that is, augmenting the use of the most prevalent means of communication in these settings, that is, word of mouth communication. These could include adoption of peer-education health clubs that have been proven to be effective in similar settings in other countries, such as the five-year social marketing adolescent sexual health project implemented in Cameroon, South-Africa, Botswana and Guinea, with positive effects on contraceptive use [36].

There has been a rise in annual rate of institutional deliveries in Burkina Faso, with the most significant increment of 27.3% occurring a year after the introduction of the subsidy for emergency care in 2007 [37]. However this level of coverage is not seen to translate into ensuring the dissemination of critical maternal health information, especially in the case of rural residents, as evidenced by the observation that there was only marginal difference in obstetric fistula prevention knowledge between those who had been pregnant in the past and those that had never been pregnant. This could be due to the persistent popularity of traditional birth attendants in such settings or due to missed opportunities to disseminate health messages during hospital consultations. Indeed, most of the participants of this study stated that they believed that they would not have complications during childbirth (approximately 90% of the study population]. Opportunities to engage young women should be optimally utilized to enable mainstreaming of obstetric fistula messages into routine services such as antenatal and postnatal care services. Successful integration of family planning messages have been implemented with community based maternal health services such as antenatal and postnatal counselling services in Bangladesh, whereby a mid-term evaluation of the program showed that women in the intervention areas were more likely to use modern family planning methods within 12 months of childbirth, compared to the control area (42% vs. 27%] [38,39]. 

Almost half of the females were already married, which is very similar to figures cited by UNFPA [40]. Our findings show the need to understand the unique challenges and needs of rural areas in Burkina Faso and other developing countries, as young women living in rural areas have a higher risk of obstetric fistula and as they are at marked disadvantage when it comes to health literacy. The demographic characteristics of our study are similar to data available from the 2010 national health survey for Burkina Faso. Specifically, our result corroborates the higher levels of teenage marriage and teenage pregnancy in rural areas reported in that study [17]. This finding is also corroborated by the Population Council, which reported that nearly two thirds (62%] of girls aged 20 to 24 years, living in rural areas, were married before the age of 18 [41]. This is a finding that also exposes the contradiction between situations on the ground and what the law prescribes, whereby the legal age for marriage is set at 17 years for girls in Burkina Faso [42]. In our study, 9% of young women reported that they got married by the age of 15 years, a finding that is supported by another study conducted in Burkina Faso which showed that 6% of rural girls were married under the age of 15 years [41]. This effectively signifies the importance of reaching out to girls before this age with reproductive health messages. 

While it was evident that the motorcycle was the most frequently used mode of transportation in the district, as it was relatively cheaper than a vehicle and faster than a donkey. The concern was for the women who believed an ambulance was the best mode of transport in an emergency. Only the district hospital provides ambulance services [21]. As of the conclusion of this survey, there was only one functional ambulance in the hospital. It was key to highlight this finding, as ultimately, structural improvements geared at health system strengthening need to accompany health promotion strategies to be successful.

One of the limitations of our study includes the relatively small sample size. Also, due to cluster sampling, participants in our study may be more homogeneous than the population as a whole [23]. In addition, there is potential for inter-observer bias, due to the use of more than one interviewer; a standard and intensive training was conducted for all data enumerators with a view of minimising this bias. The standard operating procedure utilized in this study also helps to reduce the risk of this form of bias.

Although this research has focused on the prevention dimension of obstetric fistula programs, we note the importance of the other two components that make up an effective fistula response, that is, treatment and reinsertion (social and economic). Slinger [43] describes the importance of a holistic approach in her commentary, where she compared obstetric fistula interventions to a tub that is filling with water; while one tries to remove the water from the bath with a cup, there is also the need to work towards closing the tap. Hence, it follows this analogy that approaches to tackling public health ills such as obstetric fistula, which have major societal ramifications, require concerted, multi-sectoral efforts that span the whole prevention–care continuum.

It is also crucial to strengthen research efforts that focus on identifying information gaps and that explore the most effective methods for disseminating information for behaviour change in health. This research has provided essential information for framing policies and designing programs to prevent obstetric fistula. Strategies must be adapted to local settings, whether urban or rural, in order to be more effective. Efforts geared towards tackling obstetric fistula should be made explicit and strengthened within the context of wider developmental objectives and global targets such as the millennium development goals whereby multi-sectoral approaches (including education, health and gender) are deployed, while harnessing the respective contributions of all stakeholders. Indeed, the eradication of obstetric fistula constitutes a human rights issue; a societal ill that has got inequality written all over it. It embodies gender inequality as well as inequality within the same feminine gender as women in rural communities are more disadvantaged compared to those in urban areas. Indeed, “in an unequal world, these women are the most unequal among unequals” [44]**.**
